# Projected phase-change memory devices

**DOI:** 10.1038/ncomms9181

**Published:** 2015-09-03

**Authors:** Wabe W. Koelmans, Abu Sebastian, Vara Prasad Jonnalagadda, Daniel Krebs, Laurent Dellmann, Evangelos Eleftheriou

**Affiliations:** 1 IBM Research—Zurich, Säumerstrasse 4, 8803 Rüschlikon, Switzerland

## Abstract

Nanoscale memory devices, whose resistance depends on the history of the electric signals applied, could become critical building blocks in new computing paradigms, such as brain-inspired computing and memcomputing. However, there are key challenges to overcome, such as the high programming power required, noise and resistance drift. Here, to address these, we present the concept of a projected memory device, whose distinguishing feature is that the physical mechanism of resistance storage is decoupled from the information-retrieval process. We designed and fabricated projected memory devices based on the phase-change storage mechanism and convincingly demonstrate the concept through detailed experimentation, supported by extensive modelling and finite-element simulations. The projected memory devices exhibit remarkably low drift and excellent noise performance. We also demonstrate active control and customization of the programming characteristics of the device that reliably realize a multitude of resistance states.

## Abstract (web summary)

Nanoscale resistive memory devices could become critical building blocks in emerging brain-inspired computing paradigms. Here, the authors demonstrate such a device—a projected memory cell—based on a phase-change material that decouples resistance storage from the information-retrieval process.

## Graphical abstract

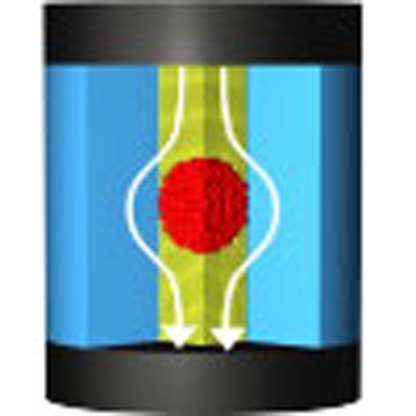

We are entering the era of cognitive computing, which holds great promise in deriving intelligence and knowledge from huge volumes of data^1^. Today's cognitive computers are based on the Von Neumann architecture, in which logic and memory are separated. Cognitive computing is inherently data-centric, and high-speed data transport between memory and processing units consumes much power. Thus, in efficient cognitive computers, logic and memory should coexist, like in brain-inspired neuromorphic computing^2^ and memcomputing^3^,^4^. The critical element in these fascinating new computing paradigms is a high-density, low-power, variable-state, programmable and non-volatile nanoscale memory device^5^,^6^,^7^,^8^,^9^,^10^. However, key challenges, such as the high programming power required, noise and resistance drift, must be overcome.

Phase-change memory devices are currently well-positioned to be used in the exploration of neuromorphic and memcomputing applications owing to the multi-level storage capability, proven large-scale manufacturability and good understanding we have of the underlying physical mechanisms and state dynamics^11^. In a conventional phase-change device, a nanometric volume of phase-change material is sandwiched between two metal electrodes. On application of electrical pulses, the phase-change material reversibly changes from the crystalline to the amorphous phase. The resistance change induced by varying the structural phase configuration is used to store information. Key in this conventional approach is that the phase-change material is used both for writing information, by undergoing a phase transition, and for retrieving the information stored, by reading its low-field electrical resistance. The drawback in this approach is that although phase-change materials have excellent phase-transition properties, that is, they can undergo phase transitions on the nanosecond timescale^12^,^13^ and down to nanoscale dimensions^12^, their highly disordered nature and high defect density make them susceptible to highly undesirable electrical effects, such as noise and drift^14^,^15^. This leads to the difficult challenge of having to optimize the phase-change properties and the electrical properties in one and the same material.

A resistive memory device concept that can overcome this crucial challenge would be a game changer for this technology. A promising step in this direction was the recent introduction of memory cells with electrically conducting surfactant layers to partially mitigate resistance drift^16^,^17^.

In this article, we expand on this idea by proposing a radical rethinking of the memory cell design, namely, the projected memory cell, and present a thorough experimental evaluation of this new concept. First, we introduce the projection concept. Next, we present the design, fabrication and simulation of projected phase-change memory devices. This is followed by experimental results, where we show almost complete elimination of drift and 1/*f* noise characteristics, thereby proving the efficacy of this concept.

## Results

### The concept of projection

**Figure 1:**
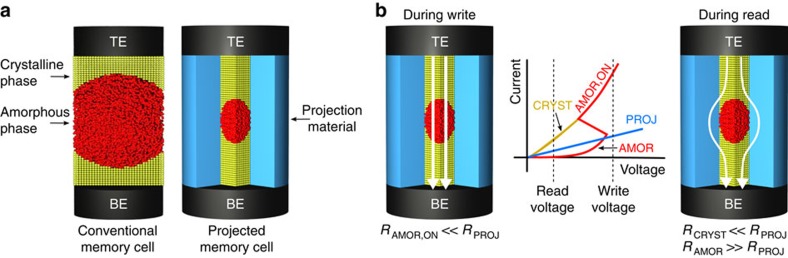
The concept of a projected memory device. (**a**) A convential phase-change memory cell (left) and a projected phase-change memory cell (right). TE and BE indicate the top and bottom electrodes, respectively. The crystalline and amorphous phases are shown schematically. The projected memory cell has an additional segment with projection material. (**b**) The desired *I*–*V* characteristics corresponding to the phase-change and projection segments are shown schematically. The phase-change section in the amorphous phase is denoted as AMOR with the corresponding resistance, *R*_AMOR_, and in the crystalline phase denoted as CRYST with resistance *R*_CRYST_. The projection segment is denoted as PROJ with resistance *R*_PROJ_. In write mode, the write voltage exceeds the threshold voltage, and the amorphous section goes into the ON-state with a resistance *R*_AMOR,ON_ that is lower than *R*_PROJ_. This ensures that most of the current flows through the phase-change segment, resulting in Joule heating and subsequent phase transition. In read mode, because *R*_PROJ_ is chosen to be much lower than *R*_AMOR_ at low fields, the current preferentially flows through the section of the projection segment that is parallel to the amorphous section. Elsewhere, the current will preferentially flow through the crystalline section.

In a projected memory device, the essential idea is to design the device in such a way that the physical mechanism of information storage is decoupled from the information-retrieval process^18^. The projected memory device or cell comprises a carefully designed segment consisting of a non-insulating material (projection segment) that is parallel to the phase-change segment (Fig. 1a). The resistance of this projection segment is judiciously chosen such that it has only a marginal influence on the write operation (during which the phase transition occurs), but a significant influence on the read operation (Fig. 1b). This is indeed possible because electrical transport in amorphous phase-change materials is highly nonlinear. At high fields, the amorphous material undergoes the so-called electronic threshold switching, leading to a low-resistive state (ON state)^19^. Thus, if during the high-field write process the resistance of the projection component is significantly higher than the ON-state resistance of the amorphous region, most of the current will flow through the phase-change segment. During the low-field read process, however, the current bypasses the highly resistive amorphous region and flows through that part of the projection segment that is parallel to it. Hence, the resistance of the device is dominated by the resistance of that part of the projection segment, and thus is a good measure of the amorphous/crystalline phase configuration. The information that typically is stored into the length of the amorphous region is in a sense projected onto the projection component. Note that even though the projection segment is present during both read and write operations, the ‘projection' is designed to occur only during the read process.

This decoupling of the information-retrieval task from the phase-change task paves the way for a complete rethinking of the memory cell design. For example, to reduce the power needed to programme a cell, the phase-change component can be made as small as or as resistive as possible, as long as the phase change (that is, the structural re-organization of atomic configurations) is facilitated. Moreover, the amorphous phase-change material can have undesirable electrical properties as long as the projection material has excellent electrical characteristics. The projection component can have very innovative geometries and resistance variations to facilitate both fabrication and multi-level storage. Furthermore, it can act as a thermal shield to prevent inter-cell interference. The cell design is guided by clear ‘design rules' under which the concept works, imposed by the highly nonlinear field-dependent electrical transport properties of phase-change materials. The projection concept is a generic concept that can be extended to certain classes of non-phase-change resistive memories, possibly in the redox class^20^,^21^.

**Figure 2:**
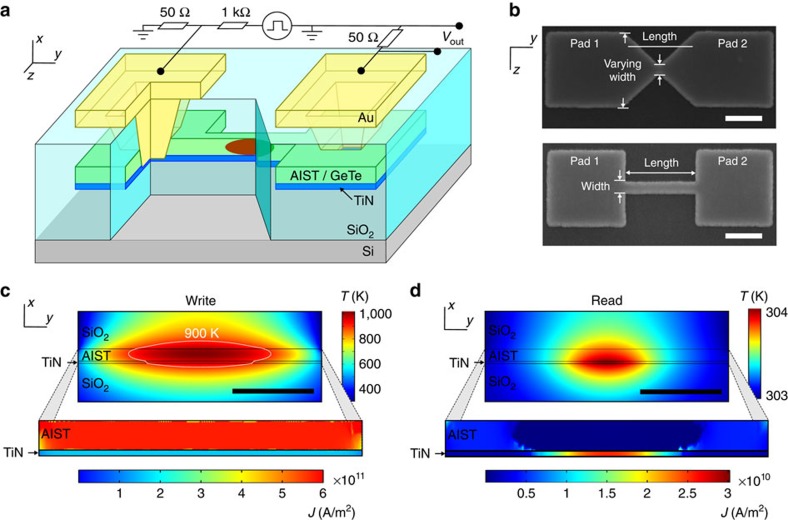
Projected memory devices. (**a**) Schematic 3D view of projected phase-change memory devices with lateral geometry. They consist of a TiN projection layer and a phase-change layer of either AIST or GeTe. These layers are surrounded by SiO_2_ for electrical and thermal insulation. The device has two electrical connections to the top level. (**b**) SEM images (top view) of a device with tapered and one with rectangular geometry during fabrication. The device length in both cases is 400 nm, and the width of the rectangular device is 50 nm. Scale bar, 200 nm. (**c**) The write process simulated by FE modelling. (top) Temperature map of the device (side view). The contour indicates the 900-K isotherm, inside which the phase-change material is assumed to be amorphous after the melt-quench process. Scale bar, 100 nm. (bottom) Current-density map (side view) during write, not drawn to scale. The current density is uniform and highest in the phase-change material. (**d**) The read process simulated by FE modelling. (top) Temperature map (side view) of the device. The temperature rise due to the read current is negligible (≈1 K). Scale bar, 100 nm. (bottom) Current-density map of the device (side view) during read, not drawn to scale. The current largely circumvents the amorphous region created by the write process of **c**, and predominantly flows through the projection component.

To demonstrate the concept of projection, several projected phase-change memory devices with a lateral cell geometry were fabricated (Fig. 2a). The lateral cell geometry was chosen for the demonstration of the concept because it offers more flexibility to explore different designs and better inspection access to the cell (during and after fabrication). However, the concept can easily be extended to commercially more viable vertical cell designs^18^. When viewed from the top, these devices possess different geometric shapes, such as tapered or rectangular (Fig. 2b). The devices consist of a 2.8-nm-thick TiN projection component and a phase-change component of either 15-nm-thick AgInSbTe (AIST) or 30-nm-thick GeTe, initially in the crystalline phase. Two different phase-change materials were used to show the versatility of the concept. Crystal growth-dominated phase-change materials GeTe^22^ and AIST^23^ were chosen to ensure the formation of well-defined amorphous regions within the crystalline matrix. Moreover, the crystalline resistivity of GeTe and AIST is advantageous for the design of cells in conjunction with a relative low-resistance projection material such as TiN. Other phase-change materials may necessitate a more resistive projection material, such as TaN.

We conducted extensive finite-element (FE) simulations to investigate the electrical and thermal characteristics of these devices. To derive the parameters for electrical transport associated with the various materials, we fabricated devices with only a projection layer or a phase-change layer and conducted field- and temperature-dependent transport measurements (see Methods). The FE simulations clearly showed the feasibility of forming substantial amorphous regions in the crystalline matrix by creating temperatures in excess of 900 K in the device, which is the typical melting temperature of phase-change materials (Fig. 2c). During writing, the current density was uniformly higher in the phase-change material than in the projection material. During reading, the amorphous region was circumvented by the current (Fig. 2d).

### Experimental results

**Figure 3:**
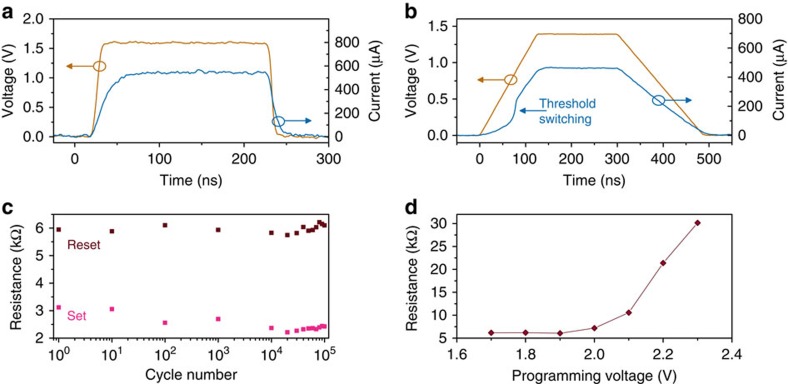
Programming of projected memory devices. (**a**) Voltage and subsequent current flowing through the device when a reset pulse is applied. The melt-quench process results in the creation of a higher resistance state that corresponds to an amorphous length of ≈30 nm. (**b**) Voltage and subsequent current when a set pulse is applied. The onset of threshold switching can clearly be seen in the initial part of the pulse. This pulse results in the crystallization of the amorphous region that was created by the reset pulse, and the resistance of the device drops to 2.6 kΩ. (**c**) Device endurance after periodic set and reset pulses. (**d**) Programming curve corresponding to a device with rectangular geometry. By increasing the amplitude of the reset pulse, the size of the amorphous region and hence the device resistance can be progressively increased to achieve almost analogue storage.

Resistive switching experiments were conducted on the projected devices. First, we present reset (increases the device resistance) and set (reduces the device resistance) experiments on a device with tapered geometry. As-fabricated, the phase-change component (AIST) was in the crystalline state with a resistance of 2.6 kΩ. A reset pulse was applied with a characteristic short trailing edge (Fig. 3a). A part of the phase-change component melted because of Joule heating, and the short trailing edge ensured a fast quench process. The resulting glass transition created an amorphous region within the crystalline matrix, which caused the device resistance to increase to 8.0 kΩ. From the simulations, we estimate that the amorphous region parallel to the projection component was about 30 nm long. Next, a set pulse with a long trailing edge was applied to crystallize. To visualize the threshold switching, we chose a set pulse that also had a long rising edge (Fig. 3b). Prior to threshold switching, the current flowing through the device was substantially lower, with little contribution from the amorphous region. At about 80 ns, the amorphous region underwent threshold switching, and a sharp rise in current was observed. This led to significant Joule heating and crystal growth, and the cell was brought back to 2.6 kΩ.

The device endurance was tested by applying a set of shorter pulses than those shown in Fig. 3a,b to not unnecessarily stress the device. The reset and set pulses were chosen to be 100 and 350 ns long, respectively. The resistance levels are well separated for more than 10^5^ cycles (Fig. 3c). The shape of the pulses was not changed during the endurance experiment.

Second, we present almost analogue storage in the projected memory devices. The rectangular-shaped devices are particulary well suited for this. By increasing the amplitude of the reset pulse, we progressively increased the size of the amorphous region and hence the resulting device resistance. Such a relation between the programmed resistance level and the reset pulse amplitude is typically referred to as a programming curve (Fig. 3d).

**Figure 4:**
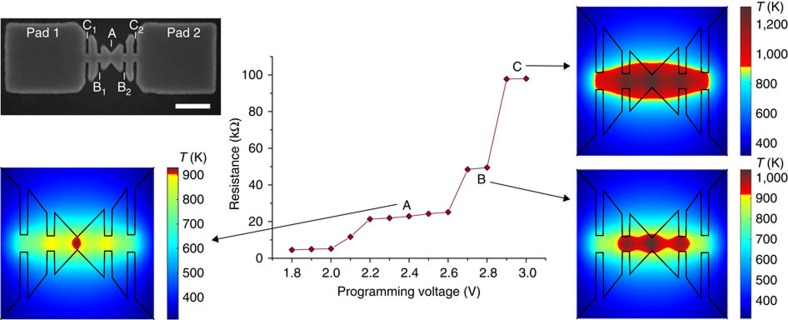
Controlled shape of the programming curve of a memory device. (top left) SEM image (top view) of the geometrically modulated memory device that has constrictions A, B_1_, B_2_, C_1_ and C_2_ to control the growth of the amorphous region. Scale bar, 200 nm. The graph shows a programming curve with distinct plateaus A, B and C caused by the constrictions. Heat maps (top view) created by FE simulation show snapshots of the phase configuration at each plateau. The yellow to red crossover at a temperature of 900 K is indicative of the crystalline to amorphous crossover.

By introducing a geometric modulation, we can influence and control the shape of the programming curve to facilitate multi-level storage. Figure 4 shows a device with spatial variations in the width. The device, containing here GeTe as the phase-change material, is characterized by several narrow constrictions designed to create hot spots and thus contain the amorphous regions. FE simulations were used to visualize the shape of the amorphous regions being created during programming. Consistent with the findings from the FE simulations, the experimental measurements showed distinct resistance plateaus in the programming curve (Fig. 4). First, the amorphous region fills the centre of the cell, as shown in the FE simulation corresponding to plateau A. If only a little more power is sent to the cell, the amorphous region grows, but will not reach the constrictions to the left (B_1_) and right (B_2_) of the centre yet. However, when the power is sufficiently high the amorphous region fills B_1_ and B_2_ at the same time. Further up, constrictions C_1_ and C_2_ will be filled, corresponding to plateau C of the graph in Fig. 4. Such programming curves are particularly well suited for multi-level storage in which distinct resistance levels need to be achieved in a highly reliable manner.

**Figure 5:**
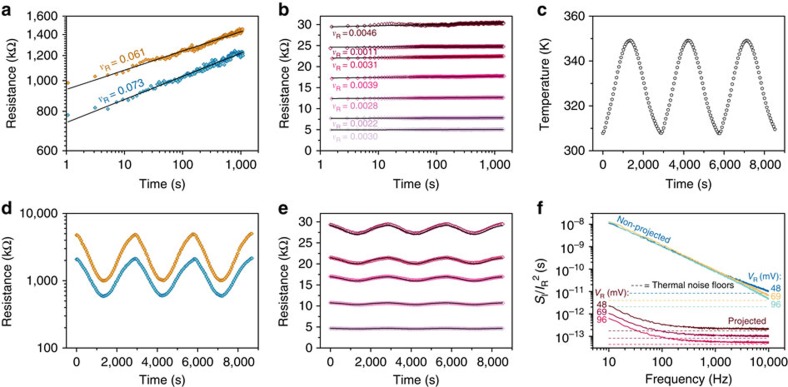
Time and temperature dependence of the resistance levels and noise characteristics. (**a**) The programmed states in a phase-change device without projection component exhibit significant temporal resistance drift at constant temperature with a drift coefficient, *ν*_R_, determined by a fit to a power law (black line), of ≈0.067 that is characteristic of the AIST phase-change material. (**b**) The programmed states in a projected memory device exhibit strongly reduced temporal resistance drift at constant ambient temperature. The black lines are fits to a power law. (**c**) Applied temperature to investigate the temperature dependence of the programmed states. (**d**) Resistance variations corresponding to two different programmed states in a non-projected memory device when the ambient temperature is varied as shown in **c**. (**e**) Resistance variations corresponding to five different programmed states in a projected memory device when the ambient temperature is varied as shown in **c**. The black lines show the resistance variation predicted by FE simulations. (**f**) Normalized spectral density of the current noise for both a projected resistive memory device and an identical phase-change device without projection component. The measurements are obtained for three different read voltages (*V*_R_). The projected memory cell is programmed to a resistance of 24 kΩ and the non-projected cell is programmed to 1.1 MΩ, both corresponding to amorphous regions of about 90 nm in length. The dashed lines show the thermal noise floors for each bias voltage and each resistance.

Next, we explore the variation in the resistance levels, a key challenge in resistive memories. To conduct a direct comparison with non-projected devices, we characterized identical lateral devices without the projection layer. The AIST-based devices were programmed to several resistance levels, and at each level the resistance of the device was monitored for 1,400 s. The temporal evolution of two programmed resistance levels in a non-projected device is shown in Fig. 5a. At constant ambient temperature, the resistance versus time is characterized by 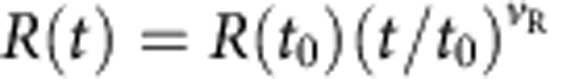, where *R*(*t*_0_) is the resistance measured at any time *t*_0_ greater than a few hundreds of nanoseconds after programming^24^,^25^. The drift coefficient, *ν*_R_, was measured to be ≈0.067, which is typical of amorphous AIST^26^. In contrast, the projected memory device exhibited a drift coefficient of only ≈0.0030 (Fig. 5b). This remarkable, 22-fold drift reduction occurs because the majority of the current flows through the projection component rather than the amorphous region.

Because electrical transport in amorphous phase-change materials is thermally activated (*E*_A_ is typically in the range of 0.2–0.4 eV), the programmed resistance levels in a conventional phase-change device exhibit a significant temperature dependence. We studied the temperature dependence of the resistance levels in both a projected memory device and a non-projected device with identical geometries. At each programmed state, we varied the ambient temperature in a sinusoidal manner between 307 and 350 K (Fig. 5c). The resulting resistance variations for the non-projected and projected memory devices are depicted in Fig. 5d,e. The resistance variations are significantly lower in the projected memory devices. In Fig. 5e, also the result of a FE simulation matching the experimental scenario is plotted. There is remarkable agreement between experiment and simulation.

Besides the time/temperature dependence of the resistance levels, another key challenge in conventional phase-change devices is that of noise. When a bias voltage, *V*_R_, was applied to read back the resistance levels, significant fluctuations on top of the mean current, *I*_R_, were observed. This relatively low-frequency noise reduces the signal-to-noise ratio and poses significant challenges for applications of phase-change devices.

We therefore also measured the normalized power spectral density of the current noise for the projected and the non-projected devices as a function of *V*_R_ (Fig. 5f). The non-projected device showed the characteristic *V*_R_ independent, and normalized current spectral density,^27^ *S*_I_/*I*_R_^2^, with a 1/*f* frequency dependence^28^ at frequencies <10 kHz, substantially higher than the thermal noise floor for a 1.1-MΩ resistor. The projected device, in contrast, exhibited a noise level comparable to the thermal noise floor of a 24-kΩ resistor and the current noise is independent of *V*_R_. In addition, the lower absolute resistance value, for the same phase configuration, allows any readout circuitry to operate at a higher bandwidth. These experiments clearly show the merits of the projection concept for countering the highly undesirable properties of resistance variations, temperature sensitivity and noise.

## Discussion

To summarize, we have presented the concept of projection in resistive memory devices, whereby the physical mechanism of storage is decoupled from the information-retrieval process. We designed, fabricated and characterized projected memory devices based on the phase-change storage mechanism in a lateral cell geometry. For the first time, the concept was convincingly demonstrated in a combination of simulations and experimental measurements. Moreover, we also showed the significant advantages such a concept could bring in overcoming the key challenges of resistance variations and noise in conventional resistive memory devices. The prospects are fascinating: this new cell architecture opens up an alternative dimension in research on resistive memory devices in the form of projection materials. A variety of metallic, semi-metallic or even semiconducting projection materials could be explored. Moreover, instead of geometric modulation of the complete device the projection segment could be designed to have spatial geometric variations as well as variations in resistivity, for example, semiconducting projection materials could be doped locally. With all these benefits, projected memory devices are likely to provide significant impetus to the field of resistive memories and could play a key role in future non-Von Neumann computing systems.

## Methods

### Device fabrication

The devices were fabricated on a silicon substrate with a thermally grown 100-nm-thick SiO_2_ top layer for thermal and electrical insulation. Next, a layer stack consisting of the projection material (2.8 nm of TiN, only for the projected memory devices), the phase-change material (15 nm of Ag_4_In_3_Sb_67_Te_26_ or 30 nm of Ge_50_Te_50_) and a capping layer (5 nm of (ZnS)_80_–(SiO_2_)_20_) was deposited. The layer stack was then patterned into the cell design desired using an e-beam-lithography process with hydrogen silsesquioxane (HSQ) resist and an ion milling step. To avoid sidewall oxidation of the phase-change material, the patterned cell was immediately passivated with 20 nm of sputtered SiO_2_. All scanning electron microscopy (SEM) images shown were recorded at this stage of the fabrication process. Electrical connections to the cell were created by first ion milling holes into the SiO_2_ passivation layer in another e-beam lithography step and then sputtering tungsten connections in a lift-off process using a third e-beam lithography step. The tungsten was then encapsulated with a layer of 60-nm-thick sputtered SiO_2_. Finally, using optical lithography and a reactive ion etch, probe pads (200 nm of sputtered gold) were connected to the tungsten.

### Simulations

Simulations using the FE method were performed with Comsol 4.3b and 5.1 (COMSOL, Inc.) using the built-in Joule heating model. The cell geometries were rebuilt in 3D from the design values and SEM images where needed. The thermal conductivities of the projection material, the insulator (SiO_2_) and the phase-change material were chosen to be 12–15, 1.4 and 2 W m^−1^ K^−1^, respectively. SiO_2_ was assumed to be a perfect insulator. To derive the parameters for electrical transport, we fabricated devices with only a projection layer (TiN) or a phase-change layer (AIST). Field- and temperature-dependent transport measurements were conducted to obtain the parameters related to electrical transport.

The TiN projection material was found to exhibit a weak temperature dependence and almost no field dependence. The resistivity of TiN as a function of temperature fits *ρ*_PM_=*ρ*_PM,0_[1+*α*_PM_(*T*−*T*_0_)], where *α*_PM_=−4.2 × 10^−4^ K^−1^, *T*_0_=303 K, *ρ*_PM,0_=3.6 × 10^−5^ Ωm and *T* denotes the temperature, see Supplementary Fig. 1. The resistance decrease with temperature is consistent with previous studies on TiN thin films at the nanometer scale^29^.

Regarding AIST, the crystalline phase exhibits a weak temperature dependence and negligible field dependence. The resistivity of c-AIST was well captured by *ρ*_c-AIST_=*ρ*_c-AIST,0_[1+*α*_c-AIST_(*T*−*T*_0_)], where *α*_c-AIST_=−5.9 × 10^−4^ K^−1^, *T*_0_=303 K and *ρ*_c-AIST,0_=9.9 × 10^−6^ Ωm, see Supplementary Fig. 2a. The resistivity of the amorphous AIST (a-AIST) material is simulated by 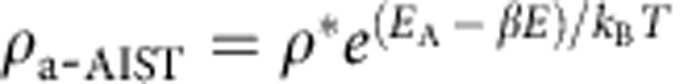, where *E* is the electric field, ρ*=1.3 × 10^−7^ Ωm, *E*_A_=0.29 eV and *β*=5.7 meV MV^−1^ m (see Supplementary Note 1 and Supplementary Figs 2b and 3a,b). Beyond a certain electric field, *E*_th_=10 MV m^−1^, the threshold-switching phenomenon will cause the resistivity of the amorphous phase to drop to a value almost identical to that of the crystalline phase, see Supplementary Fig. 3c.

Regarding GeTe, the crystalline phase exhibits a weak temperature dependence and negligible field dependence. The resistivity of c-GeTe was well captured by *ρ*_c-GeTe_=*ρ*_c-GeTe,0_[1+*α*_c-GeTe_(*T*−*T*_0_)], where *α*_c-GeTe_=4.3 × 10^−4^ K^−1^, *T*_0_=303 K and *ρ*_c-GeTe,0_=1.1 × 10^−5^ Ωm (see Supplementary Fig. 4a). The resistivity of the amorphous GeTe (a-GeTe) was well captured by 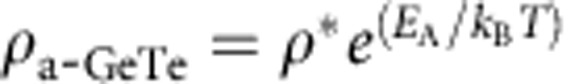, where ρ*=2.2 × 10^−6^ Ωm and *E*_A_=0.28 eV at a GeTe thickness of 30 nm (see Supplementary Fig. 4b).

### Electrical characterization

A custom-made electrical characterization platform with integrated heating stage was used for electrical measurements. The sample was mounted on an invar block with two embedded tungsten heaters. The temperature was measured using a thermocouple inserted into the invar block and controlled via a Eurotherm 2416 temperature controller. The temperature on top of the memory device chip was calibrated using an Omega silicon diode sensor. The measurement temperature was 303 K unless specified otherwise. For the annealing steps and the temperature-dependent transport measurements, the tungsten heaters were used. The ambient temperature variation experiment of Fig. 5d was performed after 150 min of annealing up to 350 K. To stabilize the resistance state, this annealing was done for each of the states programmed. Full crystallinity of the phase-change materials in the devices fabricated was verified by resistivity measurements and ensured by annealing steps if required. The devices were electrically contacted using a high-frequency Cascade Microtech Dual-Z probe. A Keithley 2400 SMU was used for DC voltage or current outputs and the measurements of the corresponding current or voltage at the device. The cell resistance was read at a constant read voltage of 0.1 V. Device programming was done using an Agilent 81150A Pulse Function Arbitrary Generator. The AC voltage and current signals were measured with a Tektronix DPO4104B oscilloscope. Mechanical relays were used to switch between AC and DC measurements.

## Additional information

**How to cite this article:** Koelmans, W.W. *et al.* Projected phase-change memory devices. *Nat. Commun.* 6:8181 doi: 10.1038/ncomms9181 (2015).

## Supplementary Material

Supplementary Information Supplementary Figures 1-4, Supplementary Note 1 and Supplementary References
