# Supplementary material for: Projected phase-change memory devices
Source: Nat Commun. 2015 Sep 3;6:8181. doi: 10.1038/ncomms9181 (PMC4569800; doi:10.1038/ncomms9181)
Supplement: Supplementary Information — Supplementary Figures 1-4, Supplementary Note 1 and Supplementary References [file ncomms9181-s1.pdf]

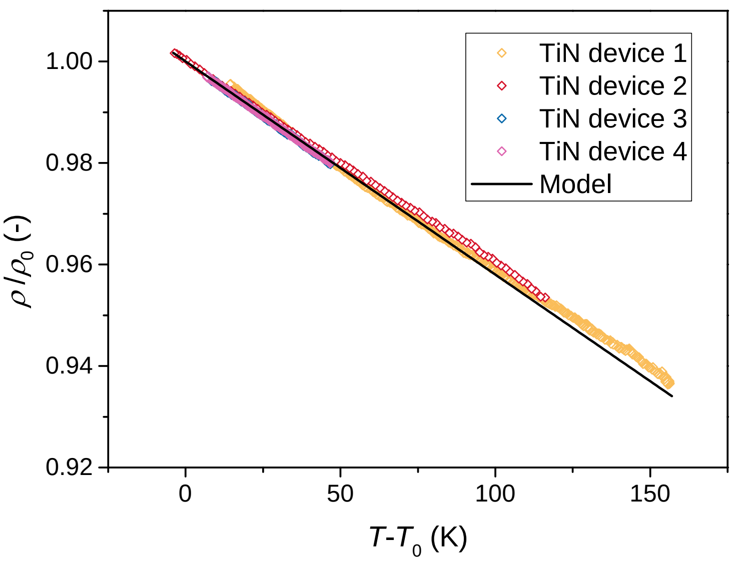


**Supplementary Figure** 1. Resistivity versus temperature measurements of TiN in four line cells. Each curve is fitted to $\rho = \rho_0 \left[1 + \alpha \left(T - T_0\right)\right]$. The mean $\alpha$ is $-4.2 \times 10^{-4}\,\mathrm{K}^{-1}$; $\rho_0 = 3.6 \times 10^{-5}\,\Omega\,\mathrm{m}$ at $2.8\,\mathrm{nm}$ thickness; with $T_0 = 303\,\mathrm{K}$. The model, plotted as a black line in the graph, uses the mean values for the parameters extracted from the fits.

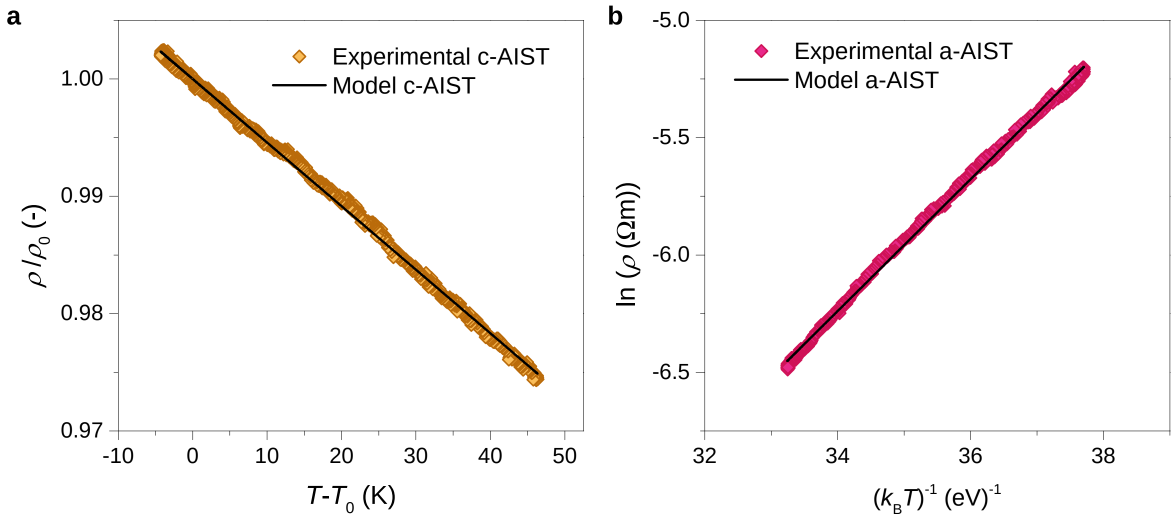


**Supplementary Figure** 2. **Temperature dependence of AIST resistivity.** **a**, Resistivity versus temperature curve of a line cell containing crystalline AIST (c-AIST). The curve is fitted to $\rho = \rho_0 \left[1 + \alpha \left(T - T_0\right)\right]$, where $\alpha$ is $-5.9 \times 10^{-4}\,\mathrm{K}^{-1}$, $T_0 = 303\,\mathrm{K}$ and $\rho = 9.9 \times 10^{-6}\,\Omega\mathrm{m}$. The black line depicts the fit. **b**, Resistivity versus temperature curve of a line cell containing amorphous AIST (a-AIST). The resisitivy $\rho$ as a function of the temperature $T$ is fitted to $\rho = \rho^{*} e^{(E_\mathrm{A}/k_\mathrm{B}T)}$, where $\rho^{*} = 1.3 \times 10^{-7}\,\Omega\mathrm{m}$ and $E_\mathrm{A} = 0.29\,\mathrm{eV}$. The black line depicts the fit.

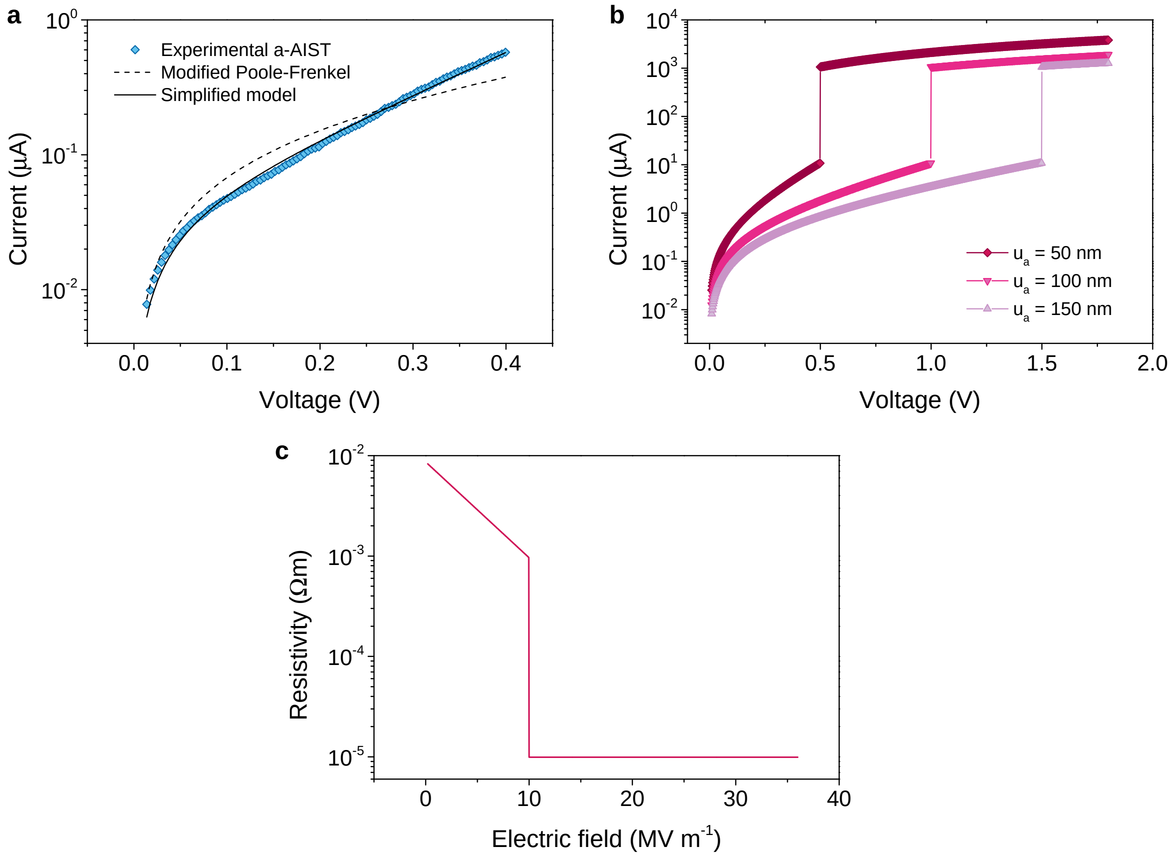


**Supplementary Figure** 3. **Field-dependent electrical transport of AIST. a**, $I-V$ characteristics of amorphous AIST. The experimental $I-V$ curve is fitted to the modified Poole–Frenkel model[1]. Also plotted is the simplified model for the resistivity $\rho$ of the amorphous AIST material as a function of the electric field $E$ and the temperature $T$: $\rho = \rho^* e^{(E_\mathrm{A}-\beta E)/k_\mathrm{B}T}$, where $\rho^* = 1.3\times10^{-7}\,\Omega\mathrm{m}$, $E_\mathrm{A} = 0.29\,\mathrm{eV}$ and $\beta = 5.7\,\mathrm{meV\,MV^{-1}\,m}$. **b**, Modelled $I-V$ characteristics of AIST with different lengths of the amorphous region, $u_\mathrm{A}$, inside a crystalline matrix. If the threshold-switching field $E_\mathrm{th}$ is reached, the materials enters into the low-resistive ON state. **c**, Model of the resistivity versus applied electric field. If $E_\mathrm{th} = 10\,\mathrm{MV\,m^{-1}}$ is reached, the material undergoes threshold switching.

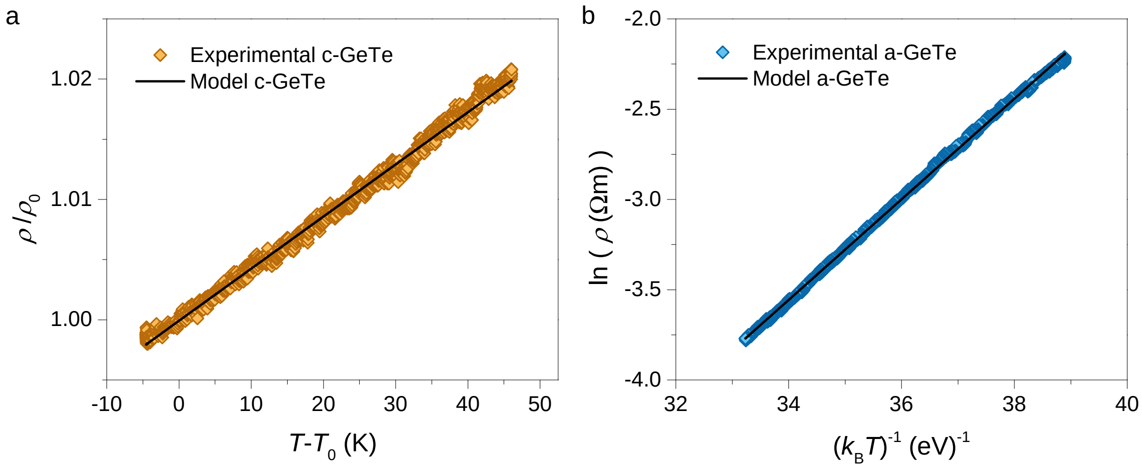


**Supplementary Figure** 4. **Temperature dependence of GeTe resistivity. a**, Resistivity versus temperature measurement of a line cell containing crystalline GeTe (c-GeTe). The resisitivy $\rho$ as a function of the temperature $T$ is fitted to $\rho = \rho_0\,[1 + \alpha\,(T - T_0)]$, where $\alpha = 4.3 \times 10^{-4}\,\mathrm{K}^{-1}$, $T_0 = 303\,\mathrm{K}$ and $\rho_0 = 1.1 \times 10^{-5}\,\Omega\mathrm{m}$. The black line depicts the fit. **b**, Resistivity versus temperature measurement of a line cell containing amorphous GeTe (a-GeTe). The resisitivy $\rho$ as a function of the temperature $T$ is fitted to $\rho = \rho^* e^{(E_\mathrm{A}/k_\mathrm{B}T)}$, where $\rho^* = 2.2 \times 10^{-6}\,\Omega\mathrm{m}$ and $E_\mathrm{A} = 0.28\,\mathrm{eV}$ at a GeTe thickness of $30\,\mathrm{nm}$. The black line depicts the fit.

## SUPPLEMENTARY NOTE 1: ELECTRICAL TRANSPORT IN AMORPHOUS AIST

In the amorphous phase, AIST exhibits a strong field- and temperature-dependence. Recent studies show that electrical transport in amorphous phase-change materials is well captured by Poole–Frenkel emission[1,2]. The Poole–Frenkel model is based on thermal emission from ionizable defect centers that are assumed to create a Coulomb potential. The key idea is that the activation energy decreases with increasing electric field. We have used a simplified model that captures the temperature dependence of both the barrier height and the carrier density: $\rho_{\text{a-AIST}} = \rho^* e^{(E_\text{A}-\beta E)/k_\text{B}T}$, where $\rho^* = 1.3 \times 10^{-7}\,\Omega\text{m}$, $E_\text{A} = 0.29\,\text{eV}$, $\beta = 5.7\,\text{meV}\,\text{MV}^{-1}\,\text{m}$ and $E$ is the electric field. The resistivity as a function of temperature for a-AIST is shown in Supplementary Fig. 2b. The experimental $I$–$V$ characteristics of the a-AIST, the Poole–Frenkel emission model[1] and our simplified model are shown in Supplementary Fig. 3a.

## SUPPLEMENTARY REFERENCES

[1]Ielmini, D. & Zhang, Y. Analytical model for subthreshold conduction and threshold switching in chalcogenide-based memory devices. *Journal of Applied Physics* **102**, 054517 (2007).

[2]Le Gallo, M., Krebs, D., Kaes, M. & Sebastian, A. Subthreshold conduction in amorphous phase-change materials. In *Proceedings of the European Phase Change and Ovonics Symposium (EPCOS)* (2014).
